# Clinical Society of the New York Polyclinic Medical School, Meeting December 4, 1905

**Published:** 1906-01

**Authors:** 


### Abstracts and Selections.

### Clinical Society of the New York Polyclinic Medical School, Meeting December 4, 1905.

SPONTANEOUS RUPTURE OF THE UTERUS DURING PREGNANCY.—Dr. L. J. Ladinski showed a uterus and adnexa which he had removed from a patient about a week before. The history given was that pregnancy followed four years after operation at the Polyclinic Hospital, and just prior to impregnation there was chronic endometritis with mattery discharge. Examination through the vagina during the fourth month of pregnancy showed the uterus to be adherent, but there was no deformity in size, form or position. Three days before operation a small blood spot was noticed, and the speaker advised absolute rest in bed and morphin. There was no further showing of blood, but the following day the patient complained of pain in her back, and two days later she collapsed. Her pulse was rapid and almost imperceptible at times, and she had intense pallor and rapid respiration. Her abdomen was distended, not in the shape of a dome, but over its entire surface. A diagnosis of ruptured abdominal pregnancy was made, and immediate operation advised. When the patient was placed on the operating table the fetus was found in its sac, floating about in the abdomen. There was a large rent in the uterus. The patient made a good recovery.

Uterine rupture during pregnancy is rare, and must not be confounded with rupture during labor. The most frequent cause for the former is the giving way of the scar of a previous Cesarian section, or of the connective tissue formed after a deep curettage. In the case described, one portion of the posterior wall of the uterus was as thin as paper. If a history of the operation performed four years before could be obtained it would assist in determining the cause of this condition.

Dr. B. Torrens said that possibly the patient was the same as one on whom he had operated about four years ago at the Polyclinic Hospital. On inserting the curette into the uterine cavity, it was found that the instrument entered the abdominal cavity through an opening in the anterior uterine wall. It was immediately withdrawn, and digital examination showed two perforations of the wall, with about one inch of connective tissue separating them. Each of the openings was large enough to admit the passage of two fingers. The uterus was packed with iodoform gauze, the cul-de-sac of Douglas was opened, and the small intestine was found adherent to the anterior uterine wall at the site of perforation. This was detached and the pelvis packed with gauze. The patient made an uneventful recovery.

Dr. R. H. M. Dawbarn said that this case reminded him of an instance in which he made a diagnosis of abdominal rupture of pregnancy, even going so far as to determine the position of the fetus. There was some bleeding from the uterus, which was enlarged and quite soft. The abdomen, when opened, allowed the escape of a very great amount of bloody material, and this being removed, it was seen that all of the viscera, the bowels especially, were covered with a new growth which proved to be sarcoma, the largest clump of which had been mistaken for the fetus.

Dermoid Cyst.—Dr. Dawbarn showed a dermoid cyst which he had removed a week previously from a girl nine years of age. The dermoid was much larger than the average specimen of its kind. There was a history of half a dozen paroxysms of pain, and when the specimen was removed the pedicle was found to be twisted upon itself a great many times. Apparently this had occurred coincidently with the pain, and, of course, had occasioned hemorrhage of the sac. The solid portion of the cyst was about the size of a small egg and was filled with teeth and bones.

